# Large and stable genome edits at the sorghum alpha kafirin locus result in changes in chromatin accessibility and globally increased expression of genes encoding lysine enrichment

**DOI:** 10.3389/fpls.2023.1116886

**Published:** 2023-03-14

**Authors:** J. Preston Hurst, Abou Yobi, Aixia Li, Shirley Sato, Thomas E. Clemente, Ruthie Angelovici, David R. Holding

**Affiliations:** ^1^ Department of Agronomy and Horticulture, University of Nebraska-Lincoln, Lincoln, NE, United States; ^2^ Department of Biological Sciences, University of Missouri, Columbia, MO, United States; ^3^ School of Life Sciences, Ministry of Education, Shandong University, Jinan, China

**Keywords:** CRISPR/Cas9, sorghum, ATACseq, RNAseq, seed protein, grain quality, lysine

## Abstract

**Introduction:**

Sorghum is a resilient and widely cultivated grain crop used for feed and food. However, it’s grain is deficient in lysine, an essential amino acid. This is due to the primary seed storage proteins, the alpha-kafirins, lacking lysine. It has been observed that reductions in alpha-kafirin protein results in rebalancing of the seed proteome and a corresponding increase in non-kafirin proteins which leads to an increased lysine content. However, the mechanisms underlying proteome rebalancing are unclear. This study characterizes a previously developed gene edited sorghum line, with deletions at the alpha kafirin locus.

**Methods:**

A single consensus guide RNA leads to tandem deletion of multiple members of the gene family in addition to the small target site mutations in remaining genes. RNA-seq and ATAC-seq were utilized to identify changes in gene expression and chromatin accessibility in developing kernels in the absence of most alpha-kafirin expression.

**Results:**

Several differentially accessible chromatin regions and differentially expressed genes were identified. Additionally, several genes upregulated in the edited sorghum line were common with their syntenic orthologues differentially expressed in maize prolamin mutants. ATAC-seq showed enrichment of the binding motif for ZmOPAQUE 11, perhaps indicating the transcription factor’s involvement in the kernel response to reduced prolamins.

**Discussion:**

Overall, this study provides a resource of genes and chromosomal regions which may be involved in sorghum’s response to reduced seed storage proteins and the process of proteome rebalancing.

## 1 Introduction

Sorghum bicolor is a grain crop used for food, animal feed and biofuel production. The fifth most important grain crop, around 60 million metric tons are harvested globally. Sorghum has superior tolerance to hot conditions, relative to rice and maize, andperforms well in water-limited conditions. These qualities hold benefit for agricultural security in developing countries as well as promise for adapting to climate resilient farming practices. Sorghum also provides a source of gluten free grain, which has utility with the increased incidence of gluten sensitivity which is rising in developed countries (Leonard et al., 2017). However, like other cereal grains, sorghum is deficient in the essential amino acid lysine (Anglani, 1998; Fantaye, 2018).

The protein fraction of sorghum grain is primarily comprised of different families of kafirin proteins, an alcohol soluble prolamin. The outer most layer of the protein body is composed of gamma-kafirins and beta-kafirins, while the inner core is composed of the alpha-kafirins (Shull et al., 1992). Of consequence to sorghum’s nutritional value, the outer protein body fraction is less digestible than the alpha-kafirin core as a result of cross-linking between kafirin species (Oria et al., 1995; Duodu et al., 2003). As a result, mutants or transgenic constructs which modify kafirins have been shown to alter protein bodies in a manner which makes them more digestible (Da Silva et al., 2011; Kumar et al., 2012; Cremer et al., 2014; Grootboom et al., 2014; Liu et al., 2019). One such kafirin allele, a characterized mutation in the alpha-kafirin signal peptide causes abnormal protein body formation and reduced alpha-kafirin accumulation (Tesso et al., 2006; Wu et al., 2013). The result is that sorghum line P721Q contains both high digestibility, from altered protein bodies, as well as high lysine, due to reduced alpha-kafirins.

Mutants such as opaque2 in maize or high digestibility, high lysine P721Q sorghum display reduced accumulation of alpha-prolamins in their grain, resulting in high lysine content (Habben et al., 1993; Wu et al., 2013). Despite a reduction in the primary storage proteins, the overall grain protein content is unchanged. The increase in lysine is caused by a change in the global composition of proteins, termed proteome rebalancing. This phenotype has potential benefits for both human nutrition and non-ruminant livestock production. However, the kernels produced by such mutants often have negative agronomic qualities, including soft kernels and susceptibility to disease, making their widespread adoption untenable. To address this in maize, breeders have introgressed modifying loci along with the *opaque2* allele, producing Quality Protein Maize (QPM). QPM kernels possess high lysine, yet the modifying alleles allow it to retain a hard endosperm (Bjarnason and Vasal, 1992; Vasal, 2000).

Despite interest in high-lysine sorghum and maize varieties, the exact cause of proteome rebalancing in the absence of prolamins is unclear. In addition to maize and sorghum, rebalancing of the proteome in response to storage protein alteration has been observed in soybean (Kinney et al., 2001; Schmidt et al., 2011), camelina (Schmidt and Pendarvis, 2017; Lyzenga et al., 2019), rice (Takaiwa et al., 2018), and wheat (Altenbach et al., 2014; Blechl et al., 2016). This suggests there may be a general adaptive pathway acting in developing seeds to allow adequate nutrition for future germination in the absence of primary storage proteins. A more comprehensive model of the biological processes leading to changes in the amino acid composition of such mutants could allow improved targets for grain protein quality improvement which circumvent the associated agronomic issues.

Previously, CRISPR/Cas9 was used to edit the alpha-kafirin gene family in sorghum variety Tx430 (Li et al., 2018). These edited lines were outcrossed to unedited, wild type Tx430, producing a set of nearly isogenic lines. Comparing the edited alpha-kafirin mutants to the wild type counterpart provides an opportunity to identify the biological changes that occur during proteome rebalancing in presence of greatly reduced alpha-kafirins. The nearly isogenic nature of these two lines reduces noise caused by differences in genomic makeup. Additionally, utilizing a reference genome for sorghum line Tx430 increases both the accuracy and total number of sequencing reads. The *Opaque2* (*o2*) mutant is the most studied prolamin mutant. Although *o2* exhibits proteome rebalancing and hence enhanced lysine, the *O2* transcription factor itself regulates a plethora of genes in its transcriptional network (Zhan et al., 2018). This feature makes it difficult to sort the signal from the noise when studying the underlying dynamics of rebalancing using an *o2* mutant. The use of a direct mutant allele of the alpha-kafirins provides an advantage in this way.

Previously, editing of alpha-kafirin gene copies produced sorghum containing increased lysine content and protein digestibility. Subsequent breeding efforts described here have produced transgene free, edited sorghum lines containing homozygous edited alleles. Using one of these lines, named 19Q4-13, we attempt to shed light on the transcriptional and chromatin changes which occur during seed development in a genome-edited sorghum variety with an edited alpha-prolamin allele. Developing kernels from the aforementioned gene-edited sorghum lines were subjected to RNA-seq and ATAC-seq (Assay for Transposase-Accessible Chromatin using sequencing), and compared to a wild type control of sorghum line Tx430. Given that the edited lines are in a Tx430 genetic background, this system provides an isogenic comparison of the regulatory changes that occur under reduced alpha-kafirin accumulation. RNA-seq was used to identify genes which may be under different transcriptional regulation under alpha-kafirin deprivation during kernel development. ATAC-seq was used to complement transcriptional analysis by identifying regions of differentially accessible chromatin.

## 2 Materials and methods

### 2.1 Transgene removal and selection of 19Q4-13

T3 sorghum lines, in a Tx430 background, carrying an edited alpha-kafirin allele (Li et al., 2018) were outcrossed to wild type Tx430. F1 plants were self-pollinated. F2 seedlings were screened for absence of the transgene carrying the editing reagentsusing PCR primers spanning the ubiquitin promoter and cas9 sequences. The kernels of F2 progeny were screened with SDS-PAGE to identify individuals with a phenotype consistent with a reduced 22 kDa alpha-kafirin and subsequent increase in non-kafirin proteins. F2:F3 line 19Q4-13 demonstrated an SDS-PAGE phenotype consistent with this expectation and was chosen for further analysis.

### 2.2 PacBio sequencing of kafirin edits

Leaf tissue was sampled from edited line 19Q4-13 and wild type Tx430 sorghum and high molecular weight DNA was extracted using Circulomics Nanobind Plant Nuclei Big DNA Kit. HiFi libraries were generated using PacBio SMRTbell Express Template Prep Kit 2.0. The libraries were sequenced on a PacBio Sequel-IIe system in HiFi-CCS mode.

Read alignment and variant calling was performed using a local assembly approach. Reads were aligned using PBMM2, NGMLR and lra. Reads which aligned to the alpha kafirin region in any alignment method were extracted and assembled into contigs using canu. The assembled local contigs were aligned to the Tx430 reference genome using lra on CONTIG mode. The contigs spanning the kafirin region was used in conjunction with MuMMER to characterize the edits induced by CRISPR/Cas9.

### 2.3 Plant material and sampling developing kernels

F3 seeds of 19Q4-13 and wild type Tx430 were planted in a greenhouse and self-pollinated. At 20 days after pollination, defined by pollen extrusion of at least 25% of the panicle, 30 kernels were sampled from throughout the seed head. Twelve total plants were sampled, six of wild-type Tx430 and six of 19Q4-13.

Fifteen of the sampled kernels were put directly into liquid nitrogen, and used for RNA extraction. The other 15 kernels were placed directly in a nuclei isolation buffer, and processed for downstream ATAC-seq.

### 2.4 Nuclei isolation and ATAC-seq

Nuclei isolation was carried out using a modified version of Parvathaneni et al., 2021’s protocol (Parvathaneni et al., 2021). Fifteen developing kernels were finely chopped with razor blades in a nuclei isolation buffer (16 mM HEPES; pH8, 200 mM sucrose, 0.8 mM MgCl2, 4 mM KCl, 32% Glycerol, 0.25% Triton X-100, 1x complete protease inhibitor, 0.1% 2-ME, 0.1 mM PMSF). The slurry was incubated on ice for twenty minutes with gentle agitation. The slurry was then filtered through four layers miracloth, twice. The resulting nuclei suspension was allocated to microcentrifuge tubes and spun at 100 G for 1 minute at 4 degrees celcius, then the supernatant transferred to another tube. This step was repeated once. These initial spins are meant to remove starches from the suspension, as the starches may interfere with Tn5 activity. A final spin was performed at 1000 RPM for fifteen minutes. The pelleted nuclei were resuspended gently in suspension buffer. Suspensions were combined into a single tube for each sample. An aliquot of each sample was stained with DAPI and placed in a hemocytometer. An estimated 500k nuclei per sample were used for tagmentation with ApexBio Tn5 mix.

Transposed DNA fragments were subject to a first round of 5 PCR cycles amplification using Illumina UDI Primers and NEBNext High-Fidelity 2x PCR Master Mix (New England Biolabs). The number of additional PCR cycles required to generate the libraries was determined by qPCR using 20x EvaGreen Dye (Biotium). The transposed amplicons were then subject to a second round of 11 PCR cycles amplification, to reach one quarter of maximum fluorescence. Bioanalyzer results showed a periodicity of DNA fragments consistent with expectations of Tn5 accessible DNA fragments. The twelve ATAC-seq libraries were loaded on an S1 flow cell and sequenced on NovaSeq 6000 instrument, 150 bp paired end.

Adaptor sequences were trimmed using NGmerge. In order to maximize read information, quality trimming was not performed. Reads were aligned using bwa-mem. Duplicate reads were discarded with Picard. Reads which aligned to the mitochondrial or chloroplast genomes were discarded. Alignments with MAPQ less than 40 were discarded.

### 2.5 RNA extraction and RNA-seq

RNA was extracted using Trizol according to manufacturer’s protocols. Twelve cDNA libraries were sequenced on one SP flow cell on NovaSeq 6000 instrument, 150 bp paired end.

### 2.6 Peak calling and DACR analysis

Peaks were called using Genrich. Peak calling was performed in two sets, one for the edited line and one for the wild type Tx430, using the six samples of each line as replicates. Peaks were kept if they met an FDR cutoff of 0.01. The two peak sets were combined as a comprehensive set of peaks, describing all accessible chromatin regions in the study. FeatureCounts was used to count the 5’ ends of reads in each peak of the comprehensive peak set per sample (**Supplemental Dataset 1**). The peak set read counts were compared using edgeR. Peak counts were normalized using TMM normalization and differential accessibility was determined using glmLRT test at a false discovery rate (FDR) cutoff of 0.1.

### 2.7 Differential expression analysis

RNA-seq reads were trimmed with NGmerge. Alignment was performed with HISAT2. Transcript abundance was quantified using stringtie2 (**Supplemental Dataset 2**). Differential expression analysis was performed with edgeR, using TMM normalization and glmLRT at an FDR cutoff of 0.1.

### 2.8 Amino acid composition analysis

For amino acid analysis, nine biological reps were sampled for each sorghum line. Pools of 10 mature kernels from each rep were ground into a flour. Free and protein bound amino acids were extracted and analyzed separately, according to Angelovici et al. (2013).

### 2.9 Reference genome

All analysis was performed using a sorghum reference genome of variety Tx430. We thank the Department of Energy Joint Genome Institute and collaborators for pre-publication access to the Tx430 genome (developed under proposal 10.46936/10.25585/60001093) for our analysis.

## 3 Results

### 3.1 Selection of 19Q4-13

**Figure 1:**
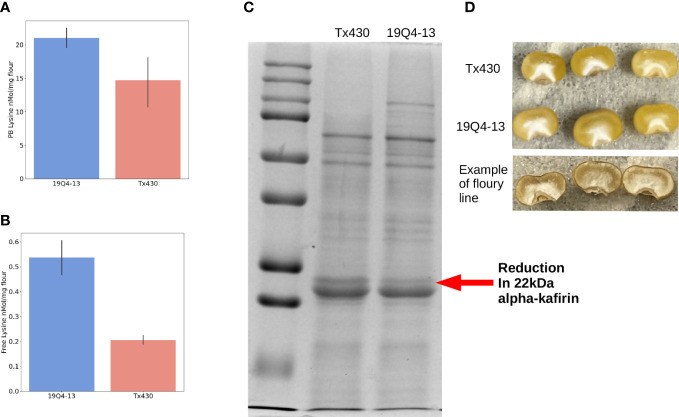
Protein phenotype of 19Q4-13. **(A)** 19Q4-13 displayed a 43% increase in protein-bound lysine content over wild-type Tx430 (p<0.05). **(B)** 19Q4-13 displayed a 161% increase in free lysine content over wild-type Tx430 (p<0.05). **(C)** The red arrow points to the 22kDa band, showing a reduction in the 22kDa alpha kafirin. **(D)** Edited line 19Q4-13 showed a similar level of kernel vitreousness to wild-type Tx430. An example of an edited sorghum line with a comparably less vitreous texture is shown below.

Many F2 families were generated from crosses between T3 plants and wild type Tx430. The T3 plants were derived from progeny of six independent transformation events, developed by Li et al. (2018). As the transformed line is in a Tx430 background, the outcrossed progeny are nearly-isogenic to Tx430. F2 seedlings which tested negative for the transgene were allowed to grow to maturity and self-pollinated. Upon visual inspection of the transgene-free F2:F3 seed on a light box, some lines were clearly segregating for the edited alpha-kafirin allele as apparent by a mixture of semi-opaque seeds and vitreous ones. Lines which displayed a homogeneous visual appearance were selected for further analysis with SDS-PAGE. After identifying lines which displayed reduced alpha-kafirin and increased non-kafirin fraction in SDS-PAGE, 19Q4-13 was selected for in depth analysis due to its semi-opaque phenotype and retention of sufficient thickness of vitreous endosperm (**Figures 1C, D**). Due to the T3 progenitors of the various lines being derived from independent editing events, phenotypic variation was observed between lines containing different edited alleles. As opposed to some lines which displayed a severely opaque and chalky phenotype, 19Q4-13 displayed a phenotype which was more opaque than wild-type yet still retained a hard endosperm. This quality holds more promise for agronomic usefulness than a line with soft kernel texture. Flour from 19Q4-13 and wild-type Tx430 were tested for amino acid composition. 19Q4-13 had a 43% increase in protein-bound lysine, from 14.7 to 21 nmol/mg flour (p less than 0.05). Free lysine increased 161%, from 0.205 to 0.536 nmol/mg flour (p less than 0.05) (**Figures 1A, B**).In addition to lysine, changes also occurred in other amino acids (**Supplemental Figure 1**). Four protein bound amino acids increased: phenylalanine, proline, serine, and tyrosine. Six free amino acids increased: alanine, arginine, asparagine, aspartate, and cysteine. Additionally, free tryptophan was decreased. These results indicate that 19Q4-13 has an edited allele that results in changes to protein composition and enhanced lysine, supporting the notion that proteome rebalancing is occurring.

### 3.2 Editing of a gene family produced complex structural variation

**Figure 2:**
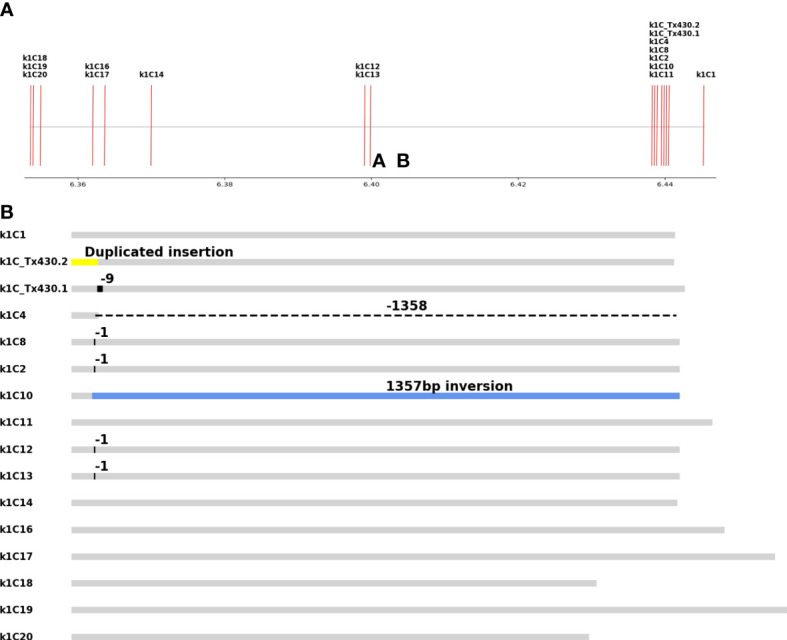
Edit characterization of 19Q4-13. **(A)** Genomic location of alpha kafirin gene copies on chromosome 5. X-axis values are chromosome 5 basepair 1x10e-7. **(B)** 8 out of 16 gene copies had an edited allele, ranging from 1bp deletions to large structural variants.

The original determination of extensive editing of alpha kafirin gene family members (K1C) from multiple Cas9 active lines was made using multiplexed Mi seq sequencing of kafirin PCR products using a degenerate K1C family primer set (Li et al., 2018). The short reads and high similarity made assigning individual K1C family member identity challenging. To unambiguously identify which K1C genes carried edits, line 19Q4-13 was subjected to WGS using Illumina 150 bp paired end reads (data unpublished). The results indicated two key pieces of information: First, short reads leave ambiguity when characterizing variants in the highly repetitive, tandem duplicated alpha kafirin region even without multiplexing. Second, the multiple gRNA binding sites across the K1C family resulted in more complex structural variation than was revealed with the PCR sequencing analysis. This was discovered after observing extreme drops in coverage adjacent to gRNA target sites. To account for these, 19Q4-13 and its Tx430, wild type counterpart were subjected to whole genome sequencing using PacBio single-molecule sequencing to ensure long reads that span multiple k1C genes (**Figure 2A**).

Two SMRT Bell libraries were constructed and sequenced to an average of 1,039,399 reads of average read length 8,515bp. After alignment, reads located between 63.6Mb and 64.6Mb on chromosome 5 were extracted and assembled into contigs using canu. Contig alignment showed that 8 of 16 alpha kafirin gene copies had a modification in 19Q4-13 (**Figure 2B**). Two copies, k1C4 and k1C10, had 1.35kb deletion and inversion respectively. The almost exact same size of the two modifications is due to an intergenic sequence match to the gRNA located 1.35 kb away from the gene body in both genes, reflecting the tandem duplicated nature of the kafirin locus. A region with an alignment spanning 64.398Mb to 64.407 had a more complex series of structural rearrangements. The reference region and contig were aligned with Mummer which demonstrated a segment containing the inversion in k1C10 had duplicated and translocated to the gRNA target site roughly 20kb downstream. A duplicate could potentially create a novel alpha kafirin copy, however the inversion in the duplicated region ensured that the duplicated alpha kafirin was non-functional.

### 3.3 Sequencing and alignment of ATAC-seq and RNA-seq NGS data

**Figure 3:**
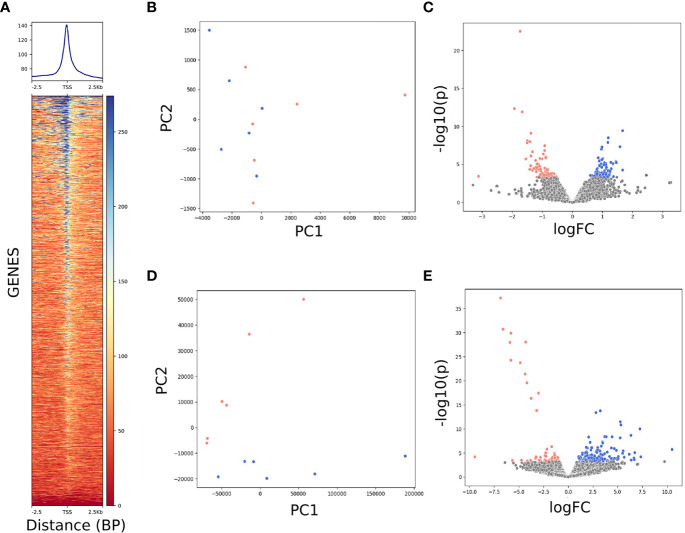
Next generation sequencing data analysis. **(A)** ATAC-seq alignment displayed an expected enrichment in reads around transcription start sites. **(B–E)** Blue represents DACR and DEGs upregulated in 19Q4-13, while pink represents those upregulated in Tx430. **(B)** ATAC-seq read counts in peaks did not appear to show clustering between Tx430 and 19Q4-13. **(C)** Volcano plot of ATAC-seq peaks. **(D)** RNA-seq expression data showed clustering between 19Q4-13 and Tx430 using the first 2 principal components. **(E)** Volcano plot of RNA-seq data.

Developing kernels, harvested 20 days after pollination, were used for nuclei isolation and RNA extraction. Isolated nuclei were subjected to Tn5 digestion and ATAC-seq library construction. The twelve ATAC-seq libraries had an average effective library size of 21.5 million read pairs. After filtering reads that aligned to the mitochondrial and chloroplast genome, 86.7% of reads were aligned. The mean insert size of aligned reads was 207.4 base pairs. Peaks were called using Genrich. In edited samples, a total of 21,843 accessible chromatin regions were identified. Wild-type samples contained 21,320 accessible regions. After merging, 32,957 regions in total were accessible. The average FRiP (Fraction of Reads in Peaks) for the ATAC-seq dataset was 12.6%, which is lower than the 20% recommended by ENCODE guidelines for human tissue. However, FRiP scores are variable between species and tissue types, thus due to a lack of ATAC-seq experiments on developing seeds it is unknown whether this value is truly low. The average TSSE (Transcription Start Site Enrichment) score was 6.5, an acceptable score regarding noise-to-signal, which backs up that the low FRiP score may be a feature of chromatin accessibility in developing kernels as opposed to a result of Tn5 over-digestion (**Figure 3A**). Future studies utilizing ATAC-seq to characterize developing kernel tissue are needed to elucidate an expected accessibility level. In total, 163 regions were differentially accessible between edited and wild type samples (**Figure 3**; **Supplemental Table 1**). The twelve RNA-seq libraries had an average library size of 76.4 million read pairs, and aligned at a rate of 91.8%. In total, 184 genes were differentially expressed (FDR<0.1) between wild-type and edited samples (**Figure 3**; **Supplemental Table 2**).

### 3.4 Deletions in alpha-kafirin genes caused alterations in chromatin accessibility and gene expression

In order to identify genes which may be regulated differently between edited and wild-type sorghum, gene models in proximity of DACR’s were pulled from the Tx430 reference genome. 326 gene models were located within 20kb of a DACR. 192 gene models were within 10kb, while 120 and 51 were within 5kb and 1kb respectively. Included in these were several of the alpha-kafirin gene copies which were edited. This could be due to changes related to the lack alpha-kafirin due to the edit, or an artifact of the duplicated segment. Alternatively, this is potentially an effect of the edits themselves preventing the normal binding of the region’s chromatin to histones.

**Figure 4:**
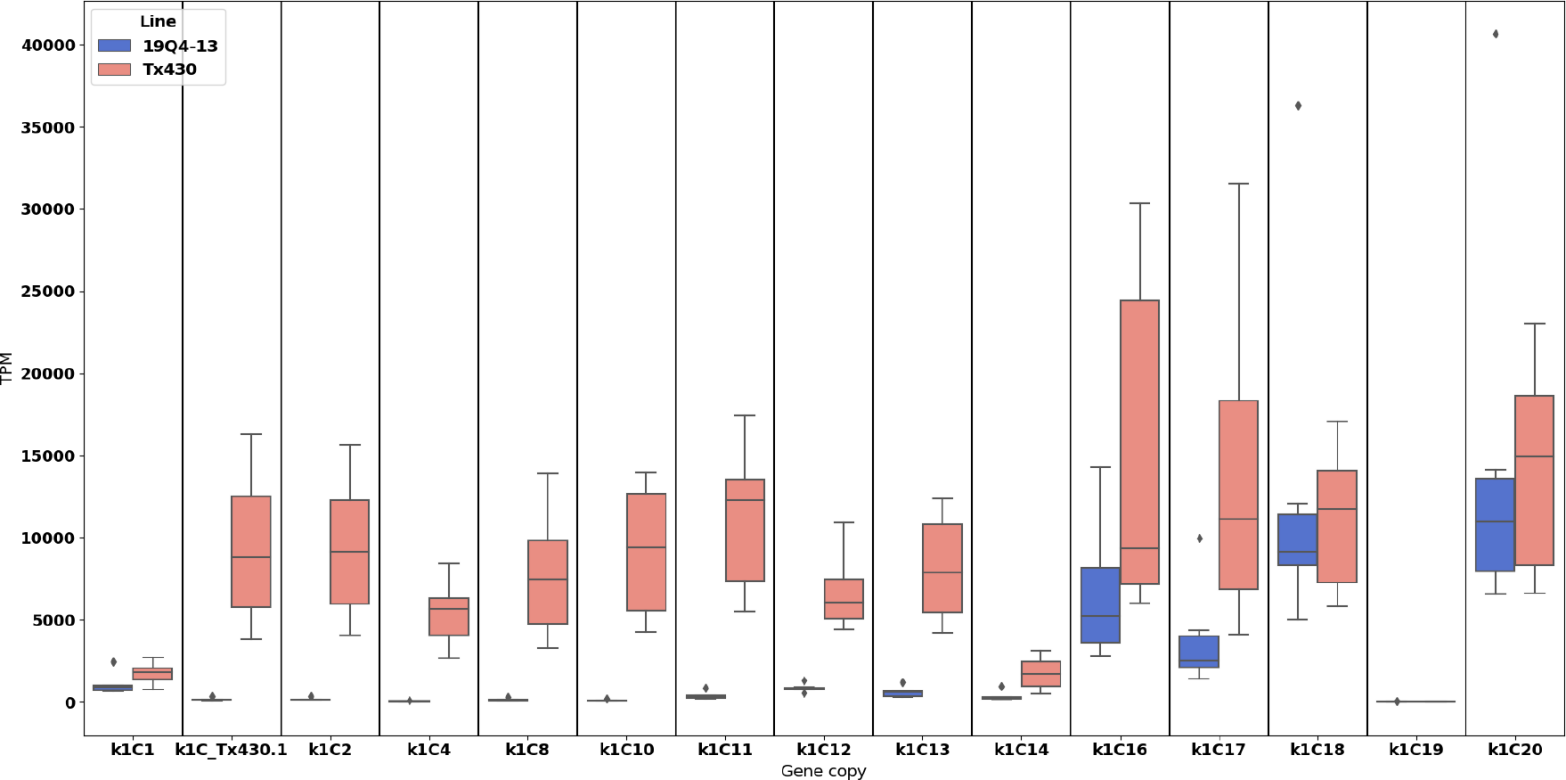
Expression differences among alpha kafirin gene copies indicates knockdown, but not knockout, of kafirin gene expression. Some kafirin gene copies which were unedited displayed reduced expression. Given the considerable structural variation, disruption in regulatory sequences may be preventing normal transcription of the unedited gene copies. TPM in the y-axis represents transcripts-per-million.

RNA-seq analysis of kernel RNA showed 184 DE genes. Of these, 109 were upregulated in the alpha-kafirin edited samples, leaving 32 genes downregulated in the edited line. Among the 32 downregulated genes were 11 were the edited alpha kafirins (**Figure 4**). In order to identify DE genes involved with the kernel protein body composition, formation and proteome rebalancing, the genes were cross referenced with the results of prior studies.

Due to the high amount of interest in the protein quality of maize, a close relative of sorghum, the sorghum syntenic orthologues of maize were extracted and compared to three studies on the maize kernel. The first, Wang et al. (2016), is a proteomic analysis of intact protein bodies from maize endosperm (Wang et al., 2016). This study identified proteins that make up the composition of kernel protein bodies in maize. A second study, Shrestha et al. (2022), performed GWAS on several kernel protein boundamino acid phenotypes in a diverse maize population (Shrestha et al., 2022). The third study, Zhan et al. (2018), performed RNA-seq and identified DE genes between wild type and o2 maize in developing endosperm (Zhan et al., 2018). Another study, Zheng et al. (2019) performed RNA-seq on developing embryos and identified differentially expressed embryonic transcripts between alpha-kafirin RNAi and WT genotypes (Zheng et al., 2019). As *o2* maize contains reduced alpha-zeins, the syntenic orthologues of alpha-kafirins, o2 maize provides a comparator to identify biologically relevant genes that may underlie proteome rebalancing and the response to prolamin knock-out in related species.

**Figure 5:**
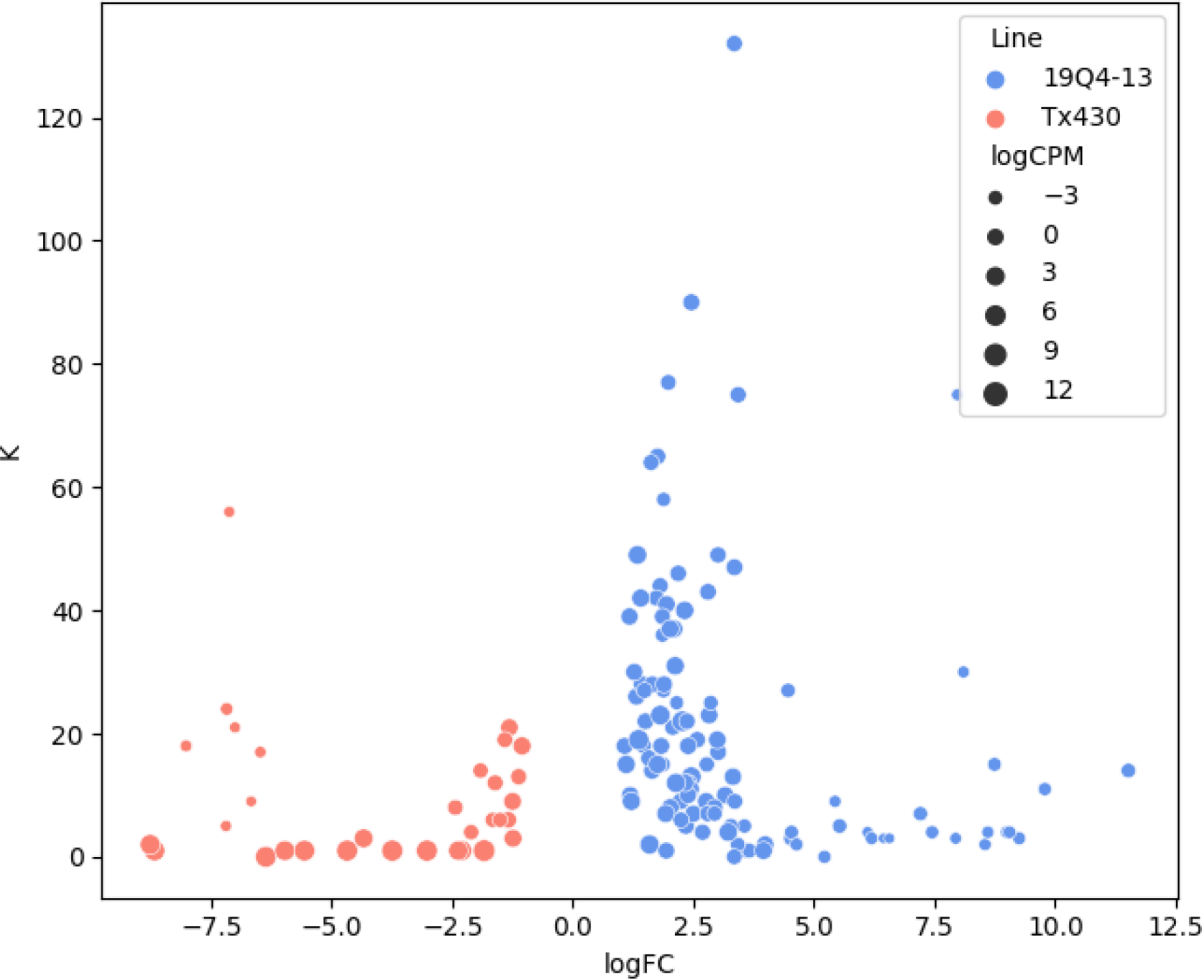
Relationship between log10 fold change and predicted lysine residues among DEGs. Genes upregulated in 19Q4-13 appear to have a higher number of predicted lysine residues than Tx430. Among DEGs, the mean number of lysine residues was 15.03 in 19Q4-13 up regulated genes versus 7.94 in genes upregulated in wild type (p=0.003). Blue indicates DEGs upregulated in 19Q4-13, while pink indicates DEGs upregulated in Tx430.

After cross referencing, 19 DE genes were implicated in at least one of the studies. One gene, SbiRTX430.02G245400, was implicated in three studies. This gene corresponds to a subtilisin protease. The syntenic orthologue was found to also be upregulated in o2 maize endosperms (Zhan et al., 2018), a component of maize protein bodies (Wang et al., 2016), as well associated with methionine to lysine ratio *via* GWAS (Shrestha et al., 2022). Interestingly, a second subtilisin protease was also upregulated in edited lines (SbiRTX430.06G174000). Three genes were implicated in two of the prior studies. SbiRTX430.01G477400, a homocysteine S-methyltransferase, was upregulated in edited sorghum as well as o2 maize endosperm (Zhan et al., 2018) and RNAi embryo (Zheng et al., 2019)). SbiRTX430.04G270300, a DUF506 domain containing protein, was upregulated in edited sorghum as well as o2 embryos (Zheng et al., 2019) and additionally, Shrestha et al’s GWAS found an association between gene proximal markers and glutamate family amino acids. Finally, a peptidase M20 family member, SbiRTX430.02G093400, was upregulated in both edited sorghum and o2 endosperm (Zhan et al., 2018) and found by proteomic analysis to be present in maize protein bodies (Wang et al., 2016). The predicted protein sequence of gene models was used to obtain a heuristic estimate of lysine residues in DEGs. Indeed, there appears to be an increase in lysine residues in the DEGs upregulated in the alpha-kafirin edited lines relative to wild-type (**Figure 5**). Among DEGs, the mean number of lysine residues was 15.03 in 19Q4-13 up regulated genes versus 7.94 in genes upregulated in wild type (p=0.003).

The DACR and DEG datasets were cross-referenced to identify differentially expressed genes which also had differentially accessible regions in proximity. Thirteen DEGs were within 10kb of a DACR, 4 of which were alpha kafirins. Interestingly, the DACR in the kafirin region was more accessible in edited lines despite the kafirins being downregulated. This is potentially due to the region having been duplicated during double strand break repair, leading to a surplus of ATAC-seq reads being aligned to the unduplicated reference region.

### 3.5 The maize opaque11 binding motif is enriched in the accessible chromatin of kafirin edited sorghum

Peaks from each line were filtered to include only those which did not overlap with its counterpart and tested for motif enrichment with STREME using the other as a control. In the edited line, nine motifs were enriched, while 19 were enriched in the wildtype samples. TOMTOM was used to identify motifs which were matched in the JASPAR core plant set. Among the nine enriched motifs in the kafirin edited lines, one matched to the *opaque11* (*o11*) binding site motif and one matched to the abscisic acid insensitive (abi) motif. Among the 19 motifs enriched in wild type, one was matched to a motif in the JASPAR core plant set, the binding site motif of maize transcription factor *O2*.

The enrichment of the o11 binding motif in edited lines prompted further investigation. A prior study, Feng et al. (2018), used CHIPseq and RNA-seq to elucidate the activity of o11 (Feng et al., 2018). Genes which were differentially expressed in o11 mutants as well genes identified as direct binding sites were cross referenced with DEGs from the current study. Twenty-two total DEGs were implicated. Five of the DEGs from the present study’s syntenic orthologues were differentially expressed in o11 mutants. Twelve DEGs were identified by Feng et al. as direct targets of *o11*. Five DEGs were identified by both RNA-seq and CHIPseq as being high-probability targets of O11. The five high probability targets were an extensin-like protein (SbiRTX430.04G038600), a beta-glycosidase related protein (SbiRTX430.06G154000), a translocon family protein (SbiRTX430.08G146200), a selenium binding protein (SbiRTX430.03G433000), and finally SbiRTX430.02G245400, a subtilisin protease. Among the five other DEGs upregulated in sorghum kafirin edits and o11 mutants were an orthologue to *sulfur-deficiency-induced-1* (*SDI1*) (SbiRTX430.01G519800) and a homocysteine S-methyltransferase(SbiRTX430.01G477400). The *SDI1* orthologue was also upregulated in *o2* embryos (Zheng et al., 2019), while the homocysteine S-methyltransferase was upregulated in both Zheng 2019 (embryo) as well as Zhan 2018 (endosperm) RNA-seq of RNAi and o2 mutants, perhaps indicating upregulation of these genes is dependent upon opacity.

## 4 Discussion

### 4.1 Editing a tandem duplicate gene family generated complex structural variation at the locus

In Li et al. (2018), the 19Q4-13 progenitor was genotyped using a sequencing by PCR approach. The results indicated a series of small insertions and deletions. However, the long read WGS approach used in the current study demonstrated the existence of large structural mutations. In hindsight, it is understandable that a PCR approach would not capture this, as the loss of primer binding sites would prevent such PCR products from being included in the sequencing reaction. Additionally, the highly repetitive nature of a tandem duplicate region impedes accurate alignment of sequencing reads. We first realized this possibility after performing WGS with 150 bp paired end Illumina sequencing gave unusually low read depth in between gRNA target sites. Long read sequencing allowed confirmation of structural variation, and also exact resolution of the breakpoints in the repetitive alpha-kafirin region. While a well known phenomena today, the research carried out in for the 2018 paper occurred during the relatively early days of using CRISPR based gene editing in crops. As such, the possibility of major structural variation was not considered. These results highlight a challenge to simultaneously editing multiple members of tandemly duplicated gene families. As CRISPR based technologies continue to advance, it will be important to consider the correct tool for specific editing applications. For example, adenine base editors, which do not induce double strand breaks, may provide less inter-target site deletions when targeting multiple loci in the same region.

### 4.2 Performance of nuclei isolation and ATAC-seq in developing kernels

ATAC-seq is used to identify regions of chromatin which are accessible to Tn5 nuclease, in order to infer locations of transcription factor binding and open chromatin. The data in this study showed comparatively low FrIP scores, yet comparatively normal TSSE scores. Potentially, the chromatin in developing sorghum kernels is relatively open compared to other tissues, leading to a wide sampling of reads across the genome. The TSSE score would indicate that over-digestion by Tn5 was not an issue, and heuristically viewing the data in IGV suggested common patterns of read depth among samples. It is known that there is hypomethylation of chromatin in the endosperm, leading to derepression of transposon expression during kernel development (Anderson and Springer, 2018). Further ATAC-seq experiments utilizing developing kernels will perhaps confirm or reject the view that developing kernel chromatin is hyperaccesible compared to other plant tissues.

Alternatively, more refined methods may be needed to properly assess chromatin in developing seeds with ATAC-seq. One challenge specific to developing seeds is how to isolate nuclei in the presence of a high amount of starch. The principal concern being the behavior of Tn5 tagmentation in a nuclei sample containing solubilized starches. In our protocol, we attempted to minimize the amount of starch in our nuclei isolate by performing two brief centrifugation steps immediately after nuclei extraction, to loosely pellet the bulk of the starch, and carefully aspirating the supernatant of suspended nuclei. Another challenge is the diverse tissue types in a developing seed. Cereal kernels consist of multiple tissue types; including endosperm, embryo, aleurone and basal endosperm transfer layer; each with functions and transcriptional regulatory modules unique to themselves. Certainly, dissecting the kernel and sampling individual tissues would give more specific results. However, an increase in the number of libraries also increases the cost of sequencing. Additionally, sorghum kernels are small in size, making dissection difficult to achieve accurately. Maize kernels, which are much larger, may be a better candidate for such an approach.

### 4.3 Transcriptome response to reduced prolamin accumulation has some conservation between sorghum and maize

In total, 184 genes were differentially expressed between Tx430 and 19Q4-13 kernels, from across a several biological categories. GO term enrichment analysis demonstrated that there is enrichment for ‘nutrient reservoir activity’ among differentially expressed genes (FDR less than 0.0001). This is expected, as the edited alpha-kafirin genes fall into this category. However, there was one gene in this category that was not a prolamin: SbiRTX430.01G133400, which encodes a germin-like or globulin seed storage protein. It has been observed previously that a reduction in prolamins in maize results in increased levels of globulins. Indeed, it was the only DEG in the ‘nutrient reservoir activity’ category upregulated in 19Q4-13. In total, nine DEGs were related to carbohydrate metabolism. All were upregulated in 19Q4-13 with the exception of one, a glycerol-3-phosphate dehydrogenase. Upregulated genes related to carbohydrate metabolism included two glycosyl hydrolases, two beta-galactosidases, a syntenic orthologue of a maize callose-synthase, beta-amylase, beta-hexosaminidase, and trehalose-6-phosphate synthase. Importantly, the DEG upregulated in 19Q4-13 showed a trend toward higher lysine residues in their translated product than those that were downregulated. These genes are potentially responsible for the enhanced lysine content that occurs through proteome rebalancing.

As one interest of this study is proteome rebalancing as a general concept, a particular focus is placed on which differentially expressed transcripts are also observed in *o2* maize. Prior studies in closely related maize provide increased confidence in genes which may be related to proteome rebalancing brought on by the absence of prolamins. These two studies, Zhan et al., 2018 and Zheng et al., 2019, compared transcripts from isolated endosperm and isolated embryos respectively. Among such genes are a homocysteine S-methyltransferase protein and an Arabidopsis sulfur deficiency induced 1 ortholog. Additionally, related to SDI1 was a sulfate transporter, a homolog of Arabidopsis SULTR1. As homocysteine-S-methyltransferase is a key enzyme involved in synthesis of methionine, it is possible that an increased synthesis of sulfur containing methionine, as a result of changes in the proteome, is depleting available sulfate and triggering a deficiency response. Cysteine is also a sulfur containing amino acid. Non-alpha kafirins, such as the gamma and beta-kafirin, are rich in cysteine (Belton et al., 2006). Recently, Massel et al. (2023) demonstrated that editing the beta-kafirin resulted in an increase of the gamma-kafirin (Massel et al., 2023). If a similar process occurs when editing the alpha-kafirins, rebalancing of the cysteine rich non-alpha kafirins could increase demand for sulfur. Further research directions may involve comparing sulfur metabolism processes between prolamin deficient kernels and wild type ones during development. In addition, were it true that the rebalancing proteome is consuming more sulfur, a question still exists as to whether these sulfur rich proteins are also contributing to increased lysine or SDI1 regulated transcripts are being translated into lysine rich proteins, or possibly that this is a collateral response in proteome rebalancing. Nonetheless, the observation of both these genes being upregulated in two different species during response to prolamin depletion holds value in understanding proteome rebalancing. Formal proof that these enzymes are involved in proteome rebalancing, and not just mRNA changes, will require further investigation.

Two proteases were also implicated in multiple studies. One syntenic gene implicated in multiple studies was a peptidase M20, SbiRTX430.02G093400. In addition to being upregulated in 19Q4-13 and *o2* endosperm, upregulation was also observed in xtito11 mutant endosperm and the presence of the corresponding protein was found to be a constituent of maize protein bodies. Another protease, a subtilisin family member (SbiRTX430.02G093400), was upregulated in 19Q4-13, *o2* endosperm, *o11* endosperm, as well as identified as constituent of maize protein bodies. In addition, the maize syntenic orthologue was identified through CHIPseq as a direct target of O11. It has been proposed that the defining point of proteome rebalancing is not at the transcriptional level, but at the translational or post translational level (Shrestha et al., 2022). Indeed, a common set of protease enzymes increased in expression under multiple prolamin deficient mutants in multiple species may speak to such a regulation. In addition, several protein kinases were also differentially regulated in 19Q4-13 sorghum. However, if true that such factors are involved in proteome rebalancing related post translational regulation, the expression of such enzymes would still seem dependent on some form of transcriptional regulation.

### 4.4 Enrichment of the Opaque11 binding motif in alpha-kafirin mutants

The homolog of AtZHOUPI, ZmOpaque11 is an endosperm-specific bHLH transcription factor (Feng et al., 2018). Mutants of both display smaller kernels, with a shriveled appearance. The embryo and endosperm of these kernels appear to lack separation, andO11 appears to regulate genes specific to the embryo surrounding region (Feng et al., 2018). By combining ATAC-seq and RNA-seq, this study was able to add an additional layer of resolution to traditional RNA-seq analysis. The use of ATAC-seq revealed accessible chromatin enrichment of the O11 motif. The corresponding RNA-seq data, combined with results from a prior study, revealed 22 DEGs in the current study whose syntenic orthologue was implicated in the O11 study. Included in these were 5 genes which were upregulated in 19Q4-13, as well as o11 maize and also identified through CHIPseq as an O11 binding site.

One such gene, a subtilisin protease (SbiRTX430.02G245400), syntenic orthologue in maize (Zm00001eb316180) was found to be present in maize protein bodies and a candidate gene in amino acid metabolism *via* GWAS (Shrestha et al., 2022). Additionally, a second subtilase was also upregulated in 19Q4-13, SbiRTX430.06G174000. The O11 homolog in Arabidopsis, AtZHOUPI, is known to regulate subtilisins associated with the interface between the endosperm and the embryo (Moussu et al., 2017). Another high confidence O11 target whose syntenic orthologue was upregulated in 19Q4-13 was an exensin-like protein, SbiRTX430.04G038600(Zm00001eb231870). Extensin-like proteins are known to be involved with the separation of embryo and endosperm during grain filling itemoussu2017zhoupi. These observations may point to the cross-talk between embryo and endosperm as a factor of proteome rebalancing.

## 5 Conclusion

Overall, the DEG and DACR observed here provide a resource for future studies of proteome rebalancing in sorghum. The DEG upregulated in the edited sorghum line have higher predicted lysine residues, perhaps indicating the source of lysine enhancement that occurs during proteome rebalancing in kafirin deficient sorghum. Additionally, utilizing syntenic orthologues of related grasses may assist in discovering conserved functions in the seed development process. The dataset produced in this study identified several overlapping DEG between prolamin-deficient sorghum and prolamin-deficient maize. A question raised is why the O11 binding motif is enriched in the edited sorghum. Further research includes investigating the role of O11 in maize and sorghum with reduced alpha-prolamins. In addition to the findings regarding the kernel response to alpha-kafirin suppression, the study also highlighted a challenge to gene editing in tandem duplicate gene families. It should also be noted that this is a comparison of a single edited allele in a F2:F3 line. Further research using the other edited alleles developed by Li et al. (2018) can be used to test how generalizable the results are to alpha-kafirin reduction and increase confidence these results are due to alterations in kafirin editing and not artifacts of the transformation process.

## Data availability statement

The data presented in this study are deposited in NCBI, accession PRJNA939832.

## Author contributions

JH and DH designed the research and wrote the manuscript. JH performed ATAC-seq, RNA-seq, and associated analysis. AY and RA performed amino acid analysis. AL performed initial edited line development. SS and TC performed sorghum transformation. All authors contributed to the article and approved the submitted version.

## Supplementary material

Supplementary Data Sheet 1 A CSV containing the raw read counts of ATAC-seq reads for each biological replicate. Counts are defined as the number of 5’ read ends in the defined peak.

Supplementary Data Sheet 2 A CSV containing raw read counts of RNA-seq data from Stringtie.

Supplementary Table 1 A table containing DACR peaks. Includes DACR location, logFC, and FDR value.

Supplementary Table 2 A table containing DEG. Includes gene name, logFC, FDR, and annotation information.

## References

[B1] Altenbach S. B. Tanaka C. K. Allen P. V. (2014). Quantitative proteomic analysis of wheat grain proteins reveals differential effects of silencing of omega-5 gliadin genes in transgenic lines. J. Cereal Sci., 118–125. doi: 10.1016/j.jcs.2013.11.008

[B2] Anderson S. N. Springer N. M. (2018). Potential roles for transposable elements in creating imprinted expression. Curr. Opin. Genet. Dev., 8–14. doi: 10.1016/j.gde.2018.01.008 29453082

[B3] Angelovici R. Lipka A. E. Deason N. Gonzalez-Jorge S. Lin H. Cepela J.. (2013). Genome-wide analysis of branched-chain amino acid levels in arabidopsis seeds. Plant Cell, 4827–4843. doi: 10.1105/tpc.113.119370 24368787 PMC3903990

[B4] Anglani C. (1998). Sorghum for human food–a review. Plant Foods Hum. Nutr., 85–95. doi: 10.1023/A:1008065519820 9839838

[B5] Belton P. Delgadillo I. Halford N. Shewry P. (2006). Kafirin structure and functionality. J. Cereal Sci., 272–286. doi: 10.1016/j.jcs.2006.05.004

[B6] Bjarnason M. Vasal S. (1992). Breeding of quality protein maize (qpm). Plant Breed. Rev., 181–216. doi: 10.1002/9780470650363.ch7

[B7] Blechl A. Beecher B. Vensel W. Tanaka C. Altenbach S. (2016). Rna interference targeting rye secalins alters flour protein composition in a wheat variety carrying a 1bl. 1rs translocation. J. Cereal Sci., 172–180. doi: 10.1016/j.jcs.2016.01.009

[B8] Cremer J. E. Liu L. Bean S. R. Ohm J. B. Tilley M. Wilson J. D.. (2014). Impacts of kafirin allelic diversity, starch content, and protein digestibility on ethanol conversion efficiency in grain sorghum. Cereal Chem., 218–227. doi: 10.1094/CCHEM-04-13-0068-R

[B9] Da Silva L. S. Taylor J. Taylor J. R. (2011). Transgenic sorghum with altered kafirin synthesis: kafirin solubility, polymerization, and protein digestion. J. Agric. Food Chem., 9265–9270. doi: 10.1021/jf201878p 21819142

[B10] Duodu K. Taylor J. Belton P. Hamaker B. (2003). Factors affecting sorghum protein digestibility. J. Cereal Sci., 117–131. doi: 10.1016/S0733-5210(03)00016-X

[B11] Fantaye B. (2018). Genetic improvement of lysine content in sorghum: A review. J. Advan. Plant Sci. 307.

[B12] Feng F. Qi W. Lv Y. Yan S. Xu L. Yang W.. (2018). Opaque11 is a central hub of the regulatory network for maize endosperm development and nutrient metabolism. Plant Cell, 375–396. doi: 10.1105/tpc.17.00616 29436476 PMC5868688

[B13] Grootboom A. W. Mkhonza N. L. Mbambo Z. O’Kennedy M. M. Da Silva L. S. Taylor J.. (2014). Co-Suppression of synthesis of major *α*-kafirin sub-class together with *γ*-kafirin-1 and *γ*-kafirin-2 required for substantially improved protein digestibility in transgenic sorghum. Plant Cell Rep., 521–537. doi: 10.1007/s00299-013-1556-5 24442398

[B14] Habben J. E. Kirleis A. W. Larkins B. A. (1993). The origin of lysine-containing proteins in opaque-2 maize endosperm. Plant Mol. Biol., 825–838. doi: 10.1007/BF00021537 8251635

[B15] Kinney A. J. Jung R. Herman E. M. (2001). Cosuppression of the α subunits of *β*-conglycinin in transgenic soybean seeds induces the formation of endoplasmic reticulum–derived protein bodies. Plant Cell, 1165–1178. doi: 10.1105/tpc.13.5.1165 11340189 PMC135556

[B16] Kumar T. Dweikat I. Sato S. Ge Z. Nersesian N. Chen H.. (2012). Modulation of kernel storage proteins in grain sorghum (sorghum bicolor (l.) moench). Plant Biotechnol. J., 533–544. doi: 10.1111/j.1467-7652.2012.00685.x 22353344

[B17] Leonard M. M. Sapone A. Catassi C. Fasano A. (2017). Celiac disease and nonceliac gluten sensitivity: a review. Jama, 647–656. doi: 10.1001/jama.2017.9730 28810029

[B18] Li A. Jia S. Yobi A. Ge Z. Sato S. J. Zhang C.. (2018). Editing of an alpha-kafirin gene family increases, digestibility and protein quality in sorghum. Plant Physiol., 1425–1438. doi: 10.1104/pp.18.00200 29925584 PMC6084649

[B19] Liu G. Gilding E. K. Kerr E. D. Schulz B. L. Tabet B. Hamaker B. R.. (2019). Increasing protein content and digestibility in sorghum grain with a synthetic biology approach. J. Cereal Sci., 27–34. doi: 10.1016/j.jcs.2018.11.001

[B20] Lyzenga W. J. Harrington M. Bekkaoui D. Wigness M. Hegedus D. D. Rozwadowski K. L. (2019). Crispr/cas9 editing of three cruciferin c homoeologues alters the seed protein profile in camelina sativa. BMC Plant Biol., 1–16. doi: 10.1186/s12870-019-1873-0 31272394 PMC6611024

[B21] Massel K. Hintzsche J. Restall J. Kerr E. D. Schulz B. L. Godwin I. D. (2023). Crispr-knockout of *β*-kafirin in sorghum does not recapitulate the grain quality of natural mutants. Planta, 1–14. doi: 10.1007/s00425-022-04038-3 36481955

[B22] Moussu S. Doll N. M. Chamot S. Brocard L. Creff A. Fourquin C.. (2017). Zhoupi and kerberos mediate embryo/endosperm separation by promoting the formation of an extracuticular sheath at the embryo surface. Plant Cell, 1642–1656. doi: 10.1105/tpc.17.00016 28696222 PMC5559742

[B23] Oria M. P. Hamaker B. R. Shull J. M. (1995). Resistance of sorghum. alpha.-,. beta.-, and. gamma.-kafirins to pepsin digestion. J. Agric. Food Chem., 2148–2153.

[B24] Parvathaneni R. K. Kumar I. Braud M. Ozersky P. Mockler T. C. Eveland A. L. (2021). Regulatory signatures of drought response in stress resilient sorghum bicolor. bioRxiv, 2020–2008. doi: 10.1101/2020.08.07.240580

[B25] Schmidt M. A. Barbazuk W. B. Sandford M. May G. Song Z. Zhou W.. (2011). Silencing of soybean seed storage proteins results in a rebalanced protein composition preserving seed protein content without major collateral changes in the metabolome and transcriptome. Plant Physiol., 330–345. doi: 10.1104/pp.111.173807 21398260 PMC3091051

[B26] Schmidt M. A. Pendarvis K. (2017). Proteome rebalancing in transgenic camelina occurs within the enlarged proteome induced by *β*-carotene accumulation and storage protein suppression. Transgenic Res., 171–186. doi: 10.1007/s11248-016-9992-y 27771868

[B27] Shrestha V. Yobi A. Slaten M. L. Chan Y. O. Holden S. Gyawali A.. (2022). Multiomics approach reveals a role of translational machinery in shaping maize kernel amino acid composition. Plant Physiol., 111–133. doi: 10.1093/plphys/kiab390 34618082 PMC8774818

[B28] Shull J. M. Watterson J. J. Kirleis A. (1992). Purification and immunocytochemical localization of kafirins insorghum bicolor (l. moench) endosperm. Protoplasma, 64–74. doi: 10.1007/BF01379281

[B29] Takaiwa F. Yang L. Wakasa Y. Ozawa K. (2018). Compensatory rebalancing of rice prolamins by production of recombinant prolamin/bioactive peptide fusion proteins within er-derived protein bodies. Plant Cell Rep., 209–223. doi: 10.1007/s00299-017-2220-2 29075848

[B30] Tesso T. Ejeta G. Chandrashekar A. Huang C. P. Tandjung A. Lewamy M.. (2006). A novel modified endosperm texture in a mutant high-protein digestibility/high-lysine grain sorghum (sorghum bicolor (l.) moench). Cereal Chem., 194–201. doi: 10.1094/CC-83-0194

[B31] Vasal S. (2000). The quality protein maize story. Food Nutr. Bull., 445–450. doi: 10.1177/156482650002100420

[B32] Wang G. Wang G. Wang J. Du Y. Yao D. Shuai B.. (2016). Comprehensive proteomic analysis of developing protein bodies in maize (zea mays) endosperm provides novel insights into its biogenesis. J. Exp. Bot., erw396. doi: 10.1093/jxb/erw396 PMC5181578 27789589

[B33] Wu Y. Yuan L. Guo X. Holding D. R. Messing J. (2013). Mutation in the seed storage protein kafirin creates a high-value food trait in sorghum. Nat. Commun., 1–7. doi: 10.1038/ncomms3217 23948869

[B34] Zhan J. Li G. Ryu C. H. Ma C. Zhang S. Lloyd A.. (2018). Opaque-2 regulates a complex gene network associated with cell differentiation and storage functions of maize endosperm. Plant Cell, 2425–2446. doi: 10.1105/tpc.18.00392 30262552 PMC6241275

[B35] Zheng X. Li Q. Li C. An D. Xiao Q. Wang W.. (2019). Intra-kernel reallocation of proteins in maize depends on vp1-mediated scutellum development and nutrient assimilation. Plant Cell, 2613–2635. doi: 10.1105/tpc.19.00444 31530735 PMC6881121

