# Supplementary figures and images for: Large and stable genome edits at the sorghum alpha kafirin locus result in changes in chromatin accessibility and globally increased expression of genes encoding lysine enrichment
Source: Front Plant Sci. 2023 Mar 14;14:1116886. doi: 10.3389/fpls.2023.1116886 (PMC10043997; doi:10.3389/fpls.2023.1116886)

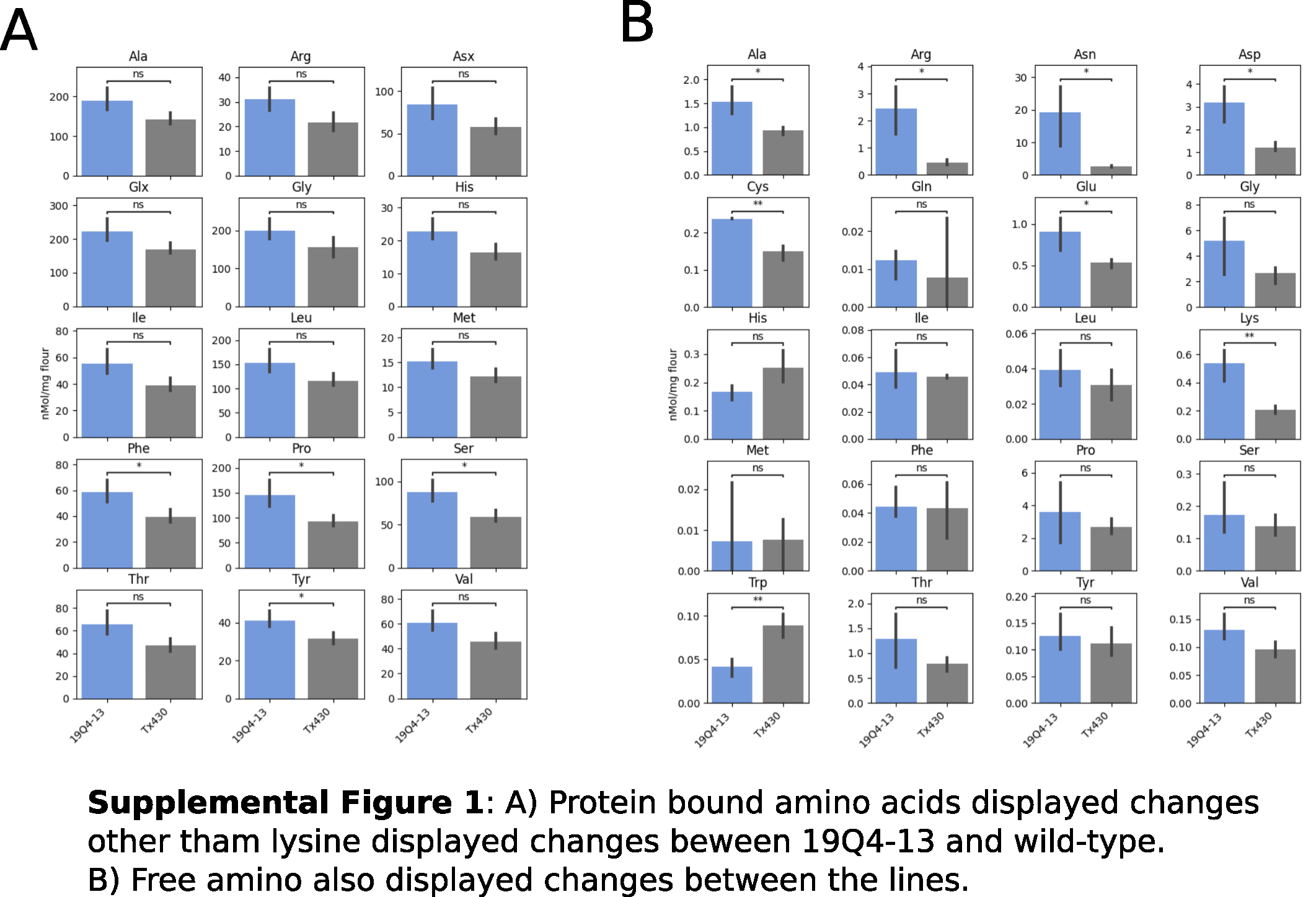

Supplement: Supplementary file 1 [file Image_1.tiff]
